# Electric multiple unit circulation plan optimization based on the branch-and-price algorithm under different maintenance management schemes

**DOI:** 10.1371/journal.pone.0199910

**Published:** 2018-07-06

**Authors:** Wenjun Li, Lei Nie, Tianwei Zhang

**Affiliations:** 1 School of Traffic and Transportation, Beijing Jiaotong University, Beijing, China; 2 School of Traffic and Transportation, Shijiazhuang Tiedao University, Shijianzhuang, China; University of Konya Technical, TURKEY

## Abstract

For railway operators, one of many important goals is to improve the utilization efficiency of electric multiple units (EMUs). When operators design EMU circulation plans, EMU type restrictions are critical factors when assigning EMUs to the correct depots for maintenance. However, existing studies only consider that EMUs are maintained at their home depots. However, targeting that problem, in this paper, an optimization model for the EMU circulation planning problem that allows depots to be selected for EMU maintenance is proposed. This model aims at optimizing the number of used EMUs and the number of EMU maintenance tasks and simultaneously incorporates other important constraints, including type restrictions, on EMU maintenance and night accommodation capacity at depots. In order to solve the model, a branch-and-price algorithm is also developed. A case study of a real-world high-speed railway was conducted to compare and analyze the effects of different maintenance location constraints. The results show that the number of EMUs used will decrease under the maintenance sharing scheme, the number of EMU maintenance tasks can be reduced, and the time occupied in EMU maintenance will be released. In addition, the scheme of maintenance resources sharing and increases to mileage limits can effectively decrease the number of EMU maintenance tasks significantly. The model and algorithm can be used as an effective quantitative analysis tool for railway operators' decision-making processes in the EMU circulation planning problem.

## 1 Introduction

China's railway enterprises have been investing huge amounts of capital in electric multiple unit (EMU) purchases of considerable scale. The cost of purchasing and maintaining the EMUs is the second greatest consumed cost after that of railway line construction. There are many difficult factors involved in EMU operations and research. At present, the operated mileage of high-speed railways has exceeded 22,000 kilometers. The number of EMUs in use has reached 2,700 standard units, and 40 active EMU depots have been put into operation, forming a high-speed railway network that includes the interregional rapid railways, inter-city railways and the existing express railway networks, which cover cities with populations exceeding 500,000. All passenger trains operating on high-speed railways in China employ EMUs. Each day, more than 5 million passengers are transported by more than 4,600 trains. In total, over 6 billion passengers have been transported. By the end of 2020, the expected operating mileage of China's high-speed railways will be 30,000 kilometers, and there will be 4,500 standard EMU units.

The continual construction of high-speed railways has brought their network characteristics into increasing prominence. Because EMUs are the most important kind of operating equipment, methods for increasing their circulation have been abundantly researched. However, the continuous development of new EMU management schemes and regulations has led to new problems that should be confronted, studied and solved in accordance with actual, changed circumstances.

Given a rail operator’s timetable on one operational day, a fleet of electric multiple units (EMUs) of different types and a rail network of routes, stations and depots, the EMU circulation planning problem refers to planning how each train on the timetable is covered by an EMU from the fleet. From the perspective of an EMU, scheduling assigns a sequence of trips as its daily workload.

At present, many scholars have conducted numerous studies on optimizing the EMU circulation problem. Hong et al. [1] proposed a two-stage approach to optimizing an EMU circulation plan while satisfying maintenance constraints. Tsuji et al. [2] proposed an algorithm that combines a local search and an ant colony algorithm to optimize an EMU circulation plan that violates a minimum number of soft constraints. Wang et al. [3] used a simulated annealing algorithm to solve EMU use maintenance program integration issues. Due to the difficulty of solving the problem with EMU maintenance, the current methods are heuristic, and using an intelligent optimization algorithm to generate a feasible solution is their main purpose. If EMU maintenance is not considered, the problem can be modeled using a multi-commodity flow model. Based on multi-commodity flow theory, Peeters and Kroon [4] modeled the train unit circulation problem as a coupling and decoupling operation and designed a branch-and-price algorithm for its solution. Cacchiani et al. [5] proposed a heuristic algorithm that can be used to quickly obtain a feasible solution. Chen et al. [6] designed a maximum-and-minimum ant colony algorithm to optimize the use of EMUs based on the guidance of an expert system. He et al. [7] established a train unit circulation plan optimization model for urban rail transit and designed a hybrid column generation algorithm to solve the model. In addition, some scholars have studied the optimization problem of robustly planning train unit circulation in emergency scenarios and emergency management problems involving EMUs in rapid-onset [8–11].

Studies of the EMU circulation planning problem have achieved a great deal, but some problems remain for further study. After EMUs are purchased, they are assigned to different affiliate railway companies. After assignment, each EMU may be used only by the affiliate company that owns it. Because of this EMU management restriction, the existing models generally assume that the EMU travels back to its home depot for maintenance, but the actual situation is that EMU maintenance can be performed at any depot that can handle that type of EMU by sharing maintenance resources if the depots cooperate with each other to maintain their EMUs. The collaborative maintenance mode must consider EMU type restrictions because the technology and maintenance tools available at each depot can maintain only EMUs of certain types. There is a research gap for this situation. Targeting this research gap, this paper focuses on studying the effects of the EMU maintenance management mode on EMU utilization efficiency. The main contributions of this study are that we propose a new optimization model that considers different EMU maintenance management modes, regulations on EMU maintenance, and each depot's capacity and EMU type restrictions, and based on the column generation method, a branch-and-price algorithm is proposed to solve the model. The influence of each maintenance management mode is analyzed numerically, and several actual cases are analyzed.

## 2 Problem description

Before a timetable is created, a line plan has been developed to define the overall train service requirements based on traveler demand and the service capacity of the infrastructure. A train timetable specifies a schedule for train operations to avoid temporal and spatial conflicts via the appropriate scheduling of trains along tracks and at stations. During the line planning stage, the train tasks in a given timetable are assigned to different companies for execution. Therefore, the trains are separated into several subsets. Each subset is the responsibility of a different affiliate company. EMUs are important moveable equipment for the train operations scheduled in a timetable. Generally, EMU depots design EMU circulation plans for operating train timetables.

(1) EMU ownership and train operations

After EMUs are purchased, they are assigned to different affiliate railway companies. The depots managed by each affiliate company assume responsibility for the EMU's operation and maintenance. After being assigned to different affiliate companies, each EMU can be used only by the affiliate company that owns it.

(2) EMU maintenance tasks and overnight accommodations

During the EMU circulation planning process, each EMU must be assigned to an appropriate depot for overnight accommodation, but the EMU must be maintained at its home depot. The daily checks and maintenance for an EMU are performed at its owner's depots. We must ensure that the maintenance and accommodation capacity of the depot is not exceeded. At present, regulations require that EMU maintenance be performed every 48 hours and every 4400 km. This EMU maintenance management mode will be referred to as the *conventional mode* in the following discussion. Under this mode, the EMU assigned to a depot can only be repaired at the home depot. Therefore, it cannot accumulate longer running times and mileage, for the maintenance is performed too often, and this limitation can simultaneously lead to problems such as (1) the potential for limitations on combinations of train connections as the EMU circulates, (2) increases in EMU maintenance workloads at EMU depots and (3) increases in maintenance costs due to the need to replace some EMU parts, which will incur large costs over the EMU’s operational time.

In this study, a mode whereby EMU maintenance resources are shared is presented. Under this mode, the related resources are not redeployed and reconfigured, but the EMUs have more choices for maintenance based on existing resource configuration. EMU maintenance can be performed at any depot while respecting the EMU type restrictions and sharing maintenance resources if the depots cooperate with each other to maintain their EMUs. In this study, only ordinary daily maintenance can be performed at any depot. However, the EMU circulation plan must ensure that each EMU returns to its home depot when it has finished circulating. An EMU may need sophisticated high-level safety maintenance at its home depot, but we do not consider the high-level maintenance arrangement. This EMU maintenance management mode will be referred to as the *collaborative mode*. When using the collaborative mode, the cooperating depots need to sign a contract that prescribes maintenance costs and prices. In accordance with the provisions of the contract, the cost of EMU maintenance can be determined. If two depots maintain EMUs for each other, they can offset each other’s costs in the settlement.

## 3 Connection network

Tables 1–3 explain the symbols of the sets, parameters, and decision variables used in the mathematical model, respectively.

**Table 1:** Set symbol definitions.

Set	Definition
***T***	is the set of trains in a given timetable, *i* ∈ ***T***
***D***	is the set of depots, *d* ∈ ***D***;
***T***_*d*_	is the set of trains that can be operated from depot *d*, *d* ∈ ***D***;
***S***	is the set of stations, *s* ∈ ***S***;
***R***	is the set of EMU maintenance routes, *r* ∈ ***R***.

**Table 2:** Parameter symbol definitions.

Parameter	Definition
*MM*	is the maintenance-based mileage limit for an EMU;
*MT*	is the maintenance-based time-interval limit for an EMU;
*P*	is a binary vector *P* = (*θ*_1_,…,*θ*_*i*_,…,*θ*_*n*_) that indicates which train nodes are covered, *i* ∈ ***T***;
*θ*_*ir*_	is a binary parameter that is equal to 1 if train *i* is covered by a route and equal to 0 otherwise, *r* ∈ ***R***;
*α*_*dr*_	is a binary parameter that is equal to 1 if an EMU goes to depot d for EMU maintenance after completing EMU maintenance route *r* and equal to 0 otherwise, *r* ∈ ***R***;
*β*_*dr*_	is a binary parameter that is equal to 1 if an EMU goes to depot d for overnight accommodation while operating on EMU maintenance route r and equal to 0 otherwise, *r* ∈ ***R***;
*u*_*dr*_	is a binary parameter that is equal to 1 if depot *d* is the origin node of maintenance route r and equal to 0 otherwise;
*v*_*dr*_	is a binary parameter that is equal to 1 if depot d is the arrival node of EMU maintenance route r and equal to 0 otherwise;
*c*_*r*_	is the cost of EMU maintenance route *r*;
*η*_*r*_	is the number of EMUs used on EMU maintenance route *r*;
*MC*_*d*_	is the EMU maintenance capacity of depot *d*, *d* ∈ ***D***;
*AC*_*d*_	is the accommodation capacity of depot *d*, *d* ∈ ***D***

**Table 3:** Decision variable symbol definition.

Decision variable	Definition
*x*_*r*_	If EMU maintenance route *r* is used in the optimal solution, *x*_*r*_ = 1, else *x*_*r*_ = 0

In this subsection, the EMU circulation planning problem is converted into an equivalent multi-depot vehicle routing problem via a connection network description. The connection network ***G*** = (***N*,*A***) is a directed graph, where ***N*** = ***T***∪***D*** is the set of nodes, and ***A*** = ***A***_0_∪***A***_1_∪***A***_2_∪***A***_3_ is the set of arcs. The arcs represent possible activities between two connected trains or potential deadheads. The arcs include connection arcs, deadhead arcs, overnight accommodation arcs and depot-station arcs.

The conditions for establishing arcs are as follows. Condition (a): The arrival station of train *i* and the origin station of train *j* are identical. Condition (b): The difference between the arrival time of train *i* and the departure time of train *j* satisfies the connection time requirements. Condition (c): The two trains use the same type of EMU. Condition (d): The two trains are operated by the same railway company.

***A***_0_ is the set of connection arcs. An arc is established in ***A***_0_ if conditions (a), (b), (c) and (d) are satisfied simultaneously. ***A***_1_ is the set of deadhead arcs. If conditions (c) and (d) are satisfied and the time required to connect two trains is sufficient, then a corresponding arc is established in this set. ***A***_2_ is the set of overnight accommodation arcs. If two trains are either at two different stations or at the same station and both conditions (c) and (d) are satisfied, a corresponding arc is established in ***A***_2_. ***A***_3_ is the set of depot-station arcs; each such arc corresponds to an EMU that is sent from the arrival station s to depot *d* after completing a train task, provided that it satisfies the EMU type restrictions at depot *d*.

Once all arcs have been established, the connection network ***G*** is complete. ***G*** can be divided into several sub-networks that are interconnected with each other through depot nodes.

**Fig 1:**
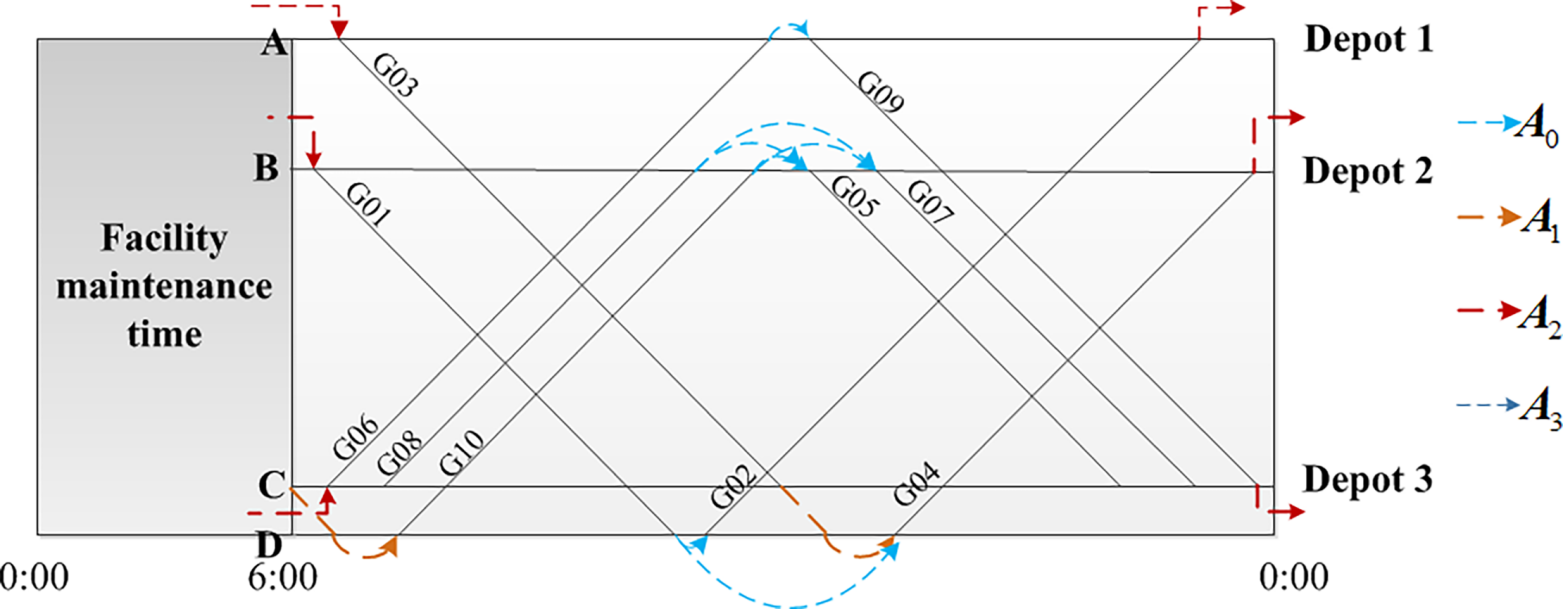
Connection network based on the time-space diagram of a train timetable.

As an illustrative example, Fig 1 shows a time-space diagram of a train timetable. Trains are indicated by straight lines, and each line is labeled with a train number. Depot 1 and Depot 2 serve station A and station B, respectively. Depot 3 serves stations C and D. The different colored arrows correspond to arcs that represent different possible connections or activities, for example, arcs in ***A***_1_ (deadhead arcs) and ***A***_2_ (overnight accommodation arcs).

From the perspective of an EMU, scheduling assigns a sequence of trains as its daily workload. A route refers to a unique path in a railway network.

## 4 Mathematical model

EMU circulation planning problem modeling must capture the above constraints and considerations to establish an optimal model and to design a corresponding algorithm to solve the problem. The problem addressed in this paper differs from existing research questions. The definition of EMU maintenance given in this paper does not impose EMU maintenance locations. In existing studies, an EMU must return to home depot for the maintenance. However, judging from practical situations, EMUs are not always required to return to their affiliated depots for maintenance. Instead, some EMUs may be sent to other depots when they need to be maintained, as long as the plans guarantee that the EMUs can return to their affiliated depots. In this way, the time for an EMU to implement train tasks will be extended.

There are two important objectives in the EMU planning problem: the number of EMUs used and the number of EMU maintenance tasks. However, the number of EMUs used is much more highly valued than the cost of EMU maintenance. Therefore, it has a higher priority and weights than EMU maintenance in the objective function.

Before establishing a model, we first need to define decision variables. Most previous studies are based on arcs to establish a nonlinear model and then use heuristic or intelligent optimization algorithms to directly search the solution to the problem.

This study will build a mathematical model based on route flow because the arc variable is not suitable for the actual circumstances of our EMU circulation planning problem. By not considering EMU maintenance constraints, EMU circulation planning problems can be modeled using common multi-commodity flow theory. However, maintenance constraints, known as resource constraints, are the hardest constraints in this study. Resource constraints limit the amounts of certain types of resources within given ranges consumed along the path to be traveled. The resource constraints to be considered in this study mainly include upper limitations on maintenance time and maintenance mileage. For each train node, the EMU must not violate the cumulative travel time and mileage regulations after the last maintenance and departure from the depot. Because maintenance or resource constraints are used to impose cumulative effects for a range of train tasks that have been performed and resource constraints cannot be effectively expressed based on arc variables, this results in breaking the problem structure in order to use the multi-commodity flow model.

As analyzed, we express the EMU circulation problem with maintenance-based path variables. According to the path flow decomposition theory, any arc-based model can be transformed into a path-based variable description model. In order to facilitate the establishment of the model and overcome the difficulties of describing resource constraints, this study introduces path variables because resource constraints are easily considered in its expression. A viable path for an EMU is defined as it starts from an origin depot to implement a number of train tasks and concludes by arriving at a depot for EMU maintenance without violating the maintenance regulations and while meeting the EMU type(s) limited by the depot.

We can generate a number of paths and select some paths by solving the mathematical model to cover the connection network and obtain the EMU circulation plans. However, presently, the obtained plans are still not completely practicable because the EMU paths might not dictate that the EMUs return to their home depots. To solve this problem, Euler loop constraints are introduced into our model, forcing the constructed path scheme to satisfy the loop formation. The Euler loop constraints will be discussed in the model description.*1 2*

The EMU circulation planning problem has two goals: minimize the number of EMUs used and minimize the number of EMU maintenance tasks.

Decision variable *x*_*r*_ is equal to 1 if *r* is used in the optimal solution and equal to 0 otherwise.

Based on the path formulation (*PF*) in the connection network, we present the following EMU circulation planning model:

$$\left(PF\right)\;obj=\sum_{r\in R}c_{r}x_{r}=\sum_{r\in R}(w_{1}c_{r1}+w_{2}c_{r2})x_{r}$$ (1)

where *c*_*r*_ is the cost of an EMU’s circulation plan, *c*_*r1*_ represents the total time for the EMU to turn over in the connection process, *c*_*r2*_ represents the cost of an EMU maintenance task, and *w*_1_ and *w*_2_ are the weight coefficients for the two costs, respectively. Because currently in our problem, we need to reduce the EMU purchasing cost as much as possible and reduce the maintenance costs as the secondary goal, *w*_1_ is much larger than *w*_2_.

Constraint (2) requires that every train is covered by exactly one route.

$$\sum_{r\in R}\theta_{ir}x_{r}=1\;\forall i\in T$$ (2)

Constraint (2) is a set partitioning constraint. Each train in the scheduled timetable can only be covered by a single EMU.

Constraint (3) indicates that the number of EMU maintenance tasks cannot exceed the available EMU maintenance capacity of *d*.

$$\sum_{r\in R}\alpha_{dr}x_{r}\leq MC_{d}\;\forall d\in D$$ (3)

The number of EMUs that can be repaired during daily working hours is limited, and the number of EMUs assigned to the depot cannot exceed the upper limit of the capability that the section can afford.

Constraint (4) restricts the availability of overnight accommodations at depot *d*.

$$\sum_{r\in R}\beta_{dr}x_{r}\leq AC_{d}\;\forall d\in D$$ (4)

After an EMU finishes implementing its train tasks, it must stop at designated depots at night, but it cannot stop at stations or elsewhere because stations belong to different departments and are not generally responsible for EMU safety, and the accommodation capacity of each depot is limited. In order to ensure that the EMU stop is orderly, accommodation capacity is respected in this model.

In accordance with Euler’s theorem, constraint (5) requires the EMU flow to be balanced at each depot.

$$\sum_{r\in R}u_{dr}x_{r}-\sum_{r\in R}v_{dr}x_{r}=0\;\forall d\in D$$ (5)

If constraint (5) is satisfied, then an Euler tour is constructed for the return of an EMU to its home depot. Because it is based on the path description of the model, the path may not be a complete loop because it cannot guarantee that some EMUs will return to their home depots. Therefore, in the model, we introduce an Euler circuit constraint to ensure that the EMU maintenance routes construct a circuit back to the home depot.

The advantage of building a model based on path variables is that the accumulation constraint based on the resource constraint form can be easily and intuitively expressed, and some other constraints are considered by transferring them onto the path generation path. However, the transformed model will bring about the problem of solving the scale. Given a large number of paths that can be combined in a given connection network, effectively generating the paths is an extremely crucial step. A large number of existing algorithms have been studied. In this paper, we employ lessons from the related theory of elementary resource constrained shortest-path problem and design an algorithm suitable for this research. The details will be discussed in part for the dynamic programming of the pricing problem. This model is solved using the following branch-and-price algorithm.

## 5 Branch-and-price algorithm

### 5.1. Branch-and-price and column generation

In this section, a branch-and-price algorithm for solving the *PF* model is presented, which is referred to as the algorithmic framework in Lin and Kwan [12]. The branch-and-price framework [13] is a high-level framework for solving large-scale ILPs by combining branch-and-bound (BB) [14] and column generation methods [15,16]. The branch pricing algorithm applies the column generation process to each node in the branch-and-bound search tree. In a generic branch-and-bound framework, if only the BB tree’s root node is solved using column generation, then the root may not contain all the columns needed to find an optimal integer solution. Therefore, the branch-and-price algorithm must perform column generation not only at the root but also at the leaf nodes of the BB tree.

Column generation is a method of solving for the path (or several paths) with the most (or nearly the most) negative reduced cost based on the pricing sub-problem. The pricing sub-problem considers the details of the EMU routes and resolves the EMU maintenance constraints. Until the *PF* model has found the optimal solution in the existing paths, column generation is used to start a pricing sub-problem, through which the *PF* model is further optimized. The interactive process between the *PF* model and the pricing sub-problem terminates when the paths generated by the pricing sub-problem cannot improve the *PF* model over several consecutive iterations.

**Fig 2:**
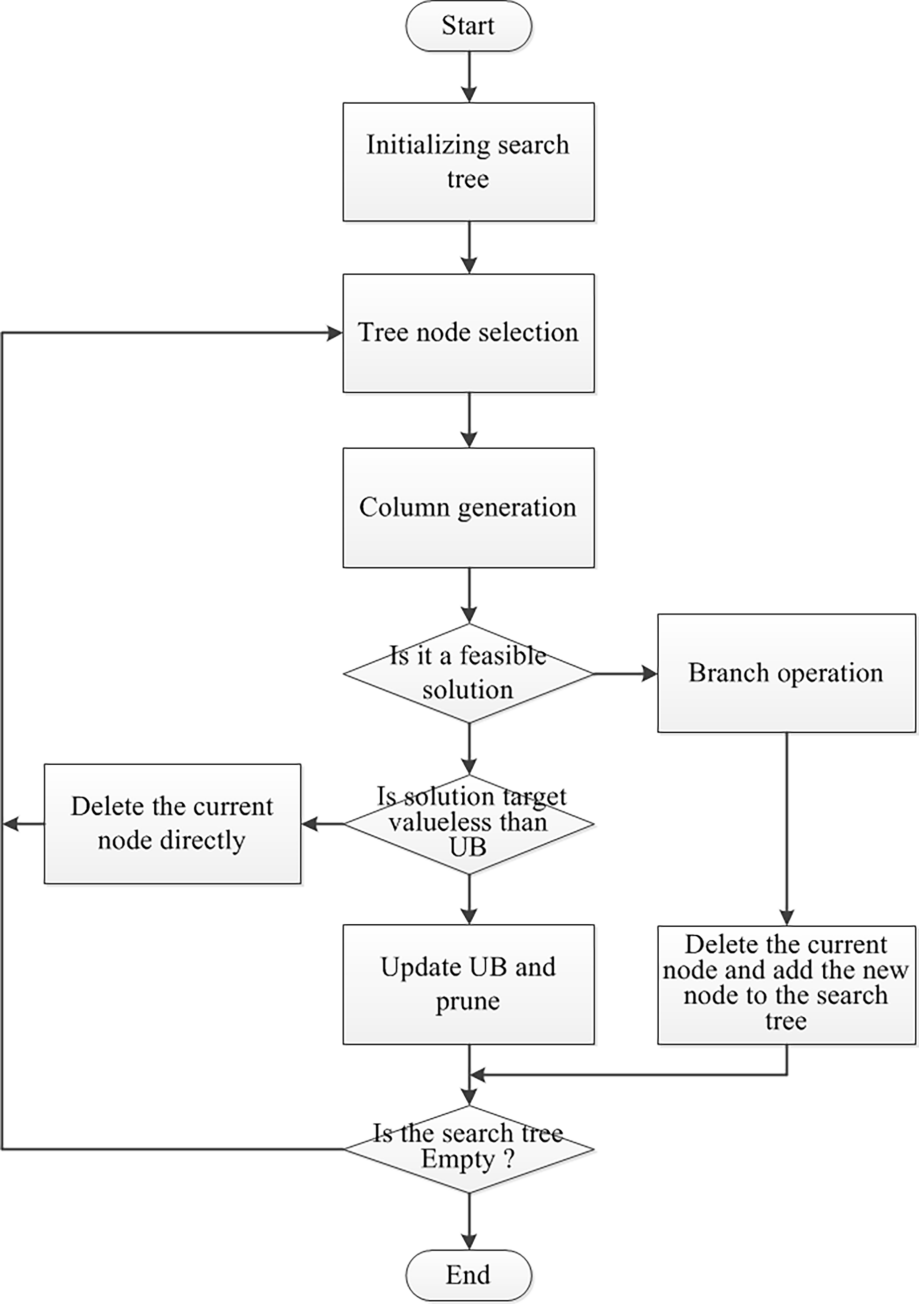
Branch-and-price algorithm flowchart.

From the above description of the branch-and-price algorithm, the process is divided into two basic levels: the outer is the branch and bound algorithm, which relaxes the integer programming problem to a linear programming problem corresponding to each node in the BB tree and searches for the fractional solution. The inner one is a column generation method, which solves the current linear relaxation problem by solving the pricing sub-problem to generate a new column for the current main problem of linear relaxation. Fig 2. below shows the flowchart of the branch-and-price algorithm.

Initializing the search tree is a very important step in the algorithm. The solution of the root node of the tree is related to the global lower bound of the outer branch-bounding, which plays a decisive role in the search process of the entire branch-and-bound tree. If we want to use the column generation method to solve the linear relaxation problem for the starting root node, we must initialize the root node, derive the corresponding dual variable value from the initial problem and pass it to the pricing sub-problem to start the column generation method. Subsequent sub-node initialization is relatively simple in the subsequent branch search process. Usually, the sub nodes inherit the feasible columns from the parent node and start their own initial solution calculation.

Another key issue of the branch-pricing algorithm is to develop effective branching strategies that should cut off the fractional solution, generate a more balanced search tree, maintain the size and depth of the search tree at a moderate level, and maintain the structure of the sub-problems along the path from the root node to the child without significantly changing the sequence of nodes solved in the problem. The ideal state is that the sub-node problem reduces the ranges of values of some variables on the basis of the parent node problem. In this way, the solution of the linear relaxation of the child nodes can be maximally consistent with the existing solution information of the parent nodes, for example, inheriting the optimal base of linear relaxation of the parent nodes to achieve the effect of a simplex warm-start and accelerate the branch pricing algorithm process.

In conventional integer programming problems, branches are usually based on variables, but this approach does not work well for the branch-and-price algorithm because it destroys the entire problem structure that disrupts the pricing process. As mentioned above, branch pricing is applied to solve a certain node of the linearly relaxed main problem. The main problem variables often correspond to a solution of the subsystems and are in the form of Boolean variables. Although the global upper bound of the main problem can be significantly improved when the variable value is set to 1 and when the variable value is determined to be 0, the column corresponding to that variable is simply deleted from the column pool of the main problem model, and that column is not allowed to be regenerated.

However, the pricing problem does not adapt to this kind of branching method. The pricing problem only concerns obtaining an optimal subsystem solution. It does not consider adopting any restriction measures in the main problem, and the pricing problem often continues to be solved according to the optimization algorithm setting, which has no effects on the entire pricing problem. In response to this problem, many scholars have conducted a large number of studies and proposed other branching strategies. Taking the vehicle routing problem as an example, it is generally possible to describe the problem model based on graph theory. The problem variables are usually defined on the arcs of the directed graphs and are then often referred to as flow variables. The main problem variables are decomposed corresponding to a path variable. Instead of branching path variables, we use flow variable branching for the main problem, which can effectively avoid the shortcomings caused by branching path variables, and decompose the original solution space into two balanced parts. One part is forbidden from passing through the arc on the directed graph, and the other one will pass the directed graph from the arc. This measure in pricing problems will reduce the search space.

### 5.2. Solution method for the pricing sub-problem

The pricing problem in this paper can be described as a problem of finding the shortest path subject to restrictions on resources:

$$\mathrm{Min}\;RC=c_{r}-\sum_{i\in \mathrm{NS}(r)}\pi_{i}\theta_{i}-\sum_{d\in D}\lambda_{d}\theta_{d}-\sum_{d\in D}\rho_{d}\alpha_{d}-\sum_{d\in D}\omega_{d}\beta_{d}.$$ (6)

In Eq (6), *RC* is the reduced cost, which is calculated using the dual variable of each constraint in the *PF* model, *π*_*i*_ corresponds to the dual variable of constraint (2), and *λ*_*d*_, *ρ*_*d*_, and *ω*_*d*_ correspond to the dual variables of constraints (3) ~ (5), respectively. This problem can be solved using the following dynamic programming algorithm. Table 4 shows the definitions of the symbols used in the algorithm.

***LAB***(*i*) represents the set of all labels associated with train node *i*, ∀*i* ∈ ***N***; *l*_*i*_ is a label *l*_*i*_ = (*RC*_*i*_,*AD*_*i*_,*AT*_*i*_,*P*_*i*_) associated with node *i* such that label $l_{i}^{h}\in \mathrm{LAB}(i)$ represents a route *r* from the starting depot *d*, to node *i*, *h* = 1, …,*H*_*i*_; *RC*_*i*_ is the reduced cost accumulated from the starting depot through node *i*; *AT*_*i*_ is the accumulated travel time from the previous EMU maintenance task to node *i*; *AD*_*i*_ is the accumulated mileage from the starting depot to node *i*; *P*_*i*_ is a binary vector recording the visited nodes, *P*_*i*_ = (*θ*_1_,*θ*_2_,…,*θ*_*n*_,…,*θ*_|*N*|_), *n* = 1, …,|***N***|, where |***N***| is the number of nodes in set *N*; and *FS*(*i*) is the set of successor nodes of *i*, *FS*(*i*) = {*j*:(*i*,*j*)*A*}.

**Table 4:** Definitions of the symbols used in the algorithm.

Symbols	Definition
*RC*	is the reduced cost, which is calculated using the dual variable of each constraint in the *PF* model;
*π*_*i*_	corresponds to the dual variable of the constraint (2);
*λ*_*d*_	correspond to the dual variables of constraints (3);
*ρ*_*d*_	correspond to the dual variables of constraints (4);
*ω*_*d*_	correspond to the dual variables of constraints (5);
*LAB*(*i*)	represents the set of all labels associated with train node *i*, ∀*i* ∈ ***N***;
*l*_*i*_	is a label *l*_*i*_ = (*RC*_*i*_,*AD*_*i*_,*AT*_*i*_,*P*_*i*_) associated with node *i* such that label $l_{i}^{h}\in \mathrm{LAB}(i)$ represents a route *r* from the starting depot *d*, to node *i*, *h* = 1, …,H*i*;
*RC*_*i*_	*RC*_*i*_ is the reduced cost accumulated from the starting depot through node *i*;
*AT*_*i*_	is the accumulated travel time from the previous EMU maintenance task to node *i*;
*AD*_*i*_	is the accumulated mileage from the starting depot to node *i*;
*P*_*i*_	is a binary vector recording the visited nodes, *P*_*i*_ = (*θ*_1_,*θ*_2_,…,*θ*_*n*_,…,*θ*_|*N*|_), *n* = 1, …,|***N***|, where |***N***| is the number of nodes in set *N*;
*FS*(*i*)	is the set of successor nodes of *i*, *FS*(*i*) = {*j*:(*i*,*j*)*A*};
*LN*	is a list in which to store nodes that have at least one label to extend.

The number of labels in the set *LAB*(*i*) is *H*_*i*_, which can be adjusted if the number of generated labels is insufficient for improving the overall problem. The solution time grows as *H*_*i*_ increases. Because the EMU circulation planning problem requires the identification of a feasible route with the minimum cost and because each route is associated with a label, the terms “**route**” and “**label**” are used interchangeably herein.

**Definition** 1. When the EMU maintenance location restriction is not under consideration, route *r* (label) is said to be feasible if *AT*_*i*_ ≤ *MT* and *AD*_*i*_ ≤ *MM* for all *i* ∈ *NS*(*r*). *NS*(*r*) is the set of nodes belonging to route *r*.

**Definition** 2. When the EMU maintenance location restriction is considered, route *r* (label) is said to be feasible if *AT*_*i*_ ≤ *MT* and *AD*_*i*_ ≤ *MM* for all *i* ∈ *NS*(*r*) and the first node and the last node are the same depot. *NS*(*r*) is the set of nodes belonging to route *r*.

**Definition** 3. Let $l_{i}^{1}=(RC_{i}^{1},AD_{i}^{1},AT_{i}^{1},P_{i}^{1})$ and $l_{i}^{2}=(RC_{i}^{2},AD_{i}^{2},AT_{i}^{2},P_{i}^{2})$ represent two labels associated with node *i* such that $RC_{i}^{1}\leq RC_{i}^{2}$, $AD_{i}^{1}\geq AD_{i}^{2}$, and $AT_{i}^{1}\leq AT_{i}^{2}$. If at least one of the inequalities is strictly satisfied, then $l_{i}^{1}$ dominates $l_{i}^{2}$.

*LN* is a list to store nodes that have at least one label to extend.

The function $Extend(l_{i}^{h},j)$ generates a feasible label *l*_*j*_ by extending *l*_*i*_ along an outgoing arc according to the resource constraints at node *j* and satisfying definitions 1 or 2 based on the EMU maintenance location restriction. If *l*_*j*_ is infeasible, then the function returns nothing. The functions *START*(*r*) and *ARRIVE*(*r*) are used to obtain the starting and ending nodes of route *r*.

The following rules control when the label stops extension under different managing situations.

1. When EMU must return to its home depot and *l*_*i*_ extends to a depot node *j*, if *START*(*r*) == *ARRIVE*(*r*)==*j*, *l*_*j*_ stops extension. Otherwise, *l*_*j*_ is deleted.
2. When the EMU can go any depot for maintenance, *l*_*i*_ extends to a depot node *j*, if *j* is a depot node which can provide appropriate maintenance service, *l*_*j*_ stops extension. Otherwise, *l*_*j*_ is deleted.

Table 5 shows the algorithm in detail.

**Table 5:** Dynamic programming labeling algorithm.

Labeling algorithm
(1) set $l_{d}^{1}=(RC_{d}^{1},AD_{d}^{1},AT_{d}^{1},p_{d}^{1})$ with $RC_{d}^{1}=0$, $AD_{d}^{1}=0$, $AT_{d}^{1}=0$, $p_{d}^{1}=(0,\ldots ,0)$ (2) $\mathrm{LAB}(d)=l_{d}^{1}\;\forall d\in D$ (3) ***LAB***(*i*) = *ϕ* ∀*i* ∈ ***T*** (4) set *LN* = *D* (5) repeat (6) select a node *i* from *LN*; (7) for each $l_{i}^{h}=(RC_{i}^{h},AD_{i}^{h},AT_{i}^{h},p_{i}^{h})\in \mathrm{LAB}(i)$ do (8) for each $j\in \mathrm{FS}(i)\&\theta_{j}^{h}<1$, where $\theta_{j}^{h}\in P_{i}^{h}$, do (9) $l_{j}\leftarrow Extend(l_{i}^{h},j)$ (10) if *l*_*i*_ is not null and *l*_*i*_ is not dominated by any label in ***LAB***(*i*) (11) set ***LAB***(*j*) = ***LAB***(*j*)∪{*l*_*i*_}; (12) remove all labels that are dominated by *l*_*i*_ from ***LAB***(*i*); (13) add node *j* to the list *LN* if *j* is not already in it; (14) end if (15) end for each (16) end for each (17) until *LN* = *ϕ* (18) return ***LAB***(*d*) for all *d* ∈ ***D***;

### 5.3 Node branch

The essence of branching is to partition the solution space by imposing mutually exclusive restrictions on each branch without losing any potential operable ones. The imposed restrictions can be implemented by explicitly discarding offending solutions instead of additional LP constraints. In a traditional BB framework, branching is generally employed to make fractional variables integral. Corresponding modifications in the sub-problem networks are made for consistency with traditional BB branching methods.

In our branching process, because the flow variables must be integral, traditional fraction-to-integer branching is needed, which is based on each individual arc. When there is a tie, the arc contained by the greatest number of paths in the *PF* solution is chosen. In forming branches, we use the method given in Alvelos [17], where a fractional arc *a* ∈ ***A*** is branched by two explicit constraints as

$$\sum_{p\in P_{a}}x_{p}\leq 0,\;\sum_{p\in P_{a}}x_{p}\geq 1$$ (7)

Constraints (7) are added where arc *a* can be deleted by removing the associated paths in the *PF* model and the associated arc in the sub-problem. *P*_*a*_ is a set of paths containing arc *a*.

### 5.4 Node selection

Node selection refers to selecting a node to solve from the active queue when the BB process must continue. We use an adaptive node selection method that combines both best-first and depth-first; it can be summarized follows: (i) complete a depth-first search; (ii) during the depth-first search, if a jump condition is triggered, then jump to an active node with the best value; and (iii) after a jump, continue with the depth-first search. More details can be found in reference [12].

## 6 Numerical experiments

In this section, we first use small cases to verify the correctness and validity of the model and the algorithm and then apply them to optimize the large-scale practical problem.

### 6.1 Small case

#### 6.1.1 EMU circulation planning in the conventional mode

**Fig 3:**
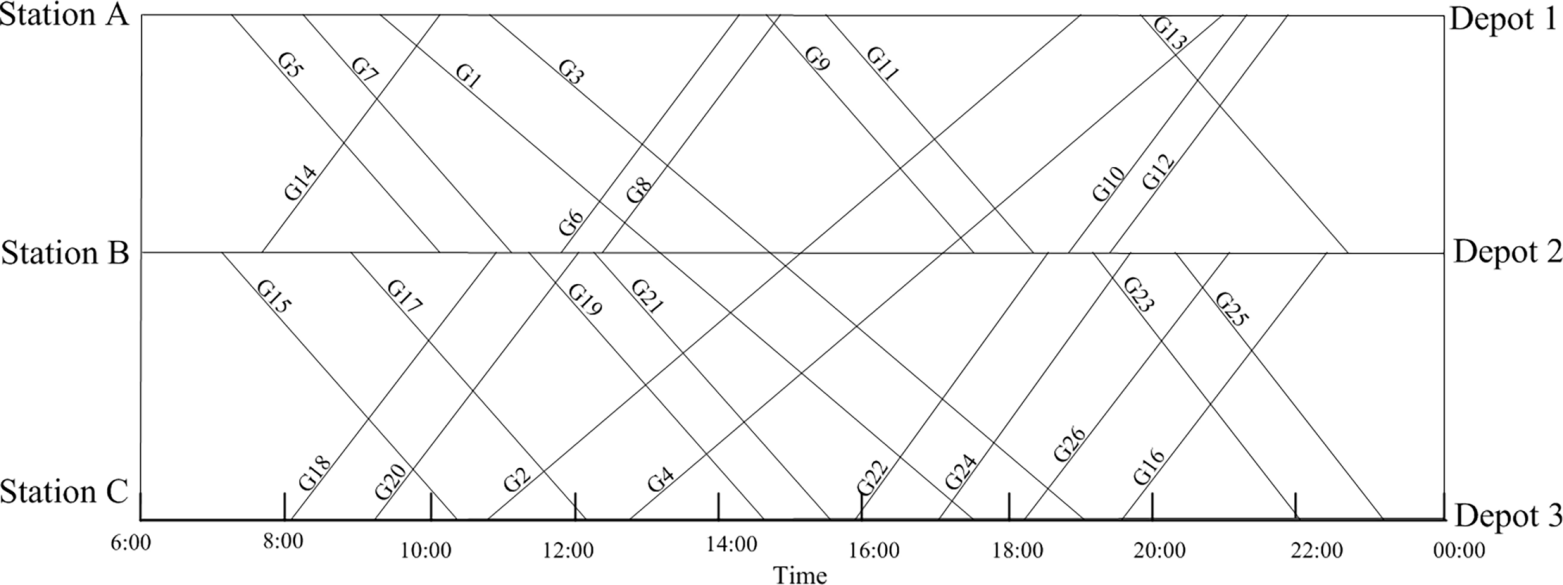
Train space-time diagram.

In order to verify the correctness, a small and simple case is studied. The specific train information is shown in Table 6 The maintenance capacity restrictions and the EMU type restrictions are neglected in this example, but the EMUs must be maintained at their home depots and cannot exceed the required maintenance intervals of 4400 km and 48 h. Fig 3 shows a space-time diagram of the given trains; the horizontal axis represents time, and the vertical axis represents the train's position. The straight lines are trajectories showing the train's location over time. Fig 3 also shows the locations of related stations and depots; A, B and C are high-speed railway stations, and depots 1, 2 and 3, which correspond to stations A, B and C, provide EMU maintenance.

**Table 6:** Train information for the simple case.

Train number	Origin station	Destination station	Departure time	Arrival time	EMU type	Travel distance (km)
G1	A	C	08:15	17:02	CRH380BL	1090
G2	C	A	09:55	18:50	CRH380BL	1090
G3	A	C	09:55	18:55	CRH380BL	1090
G4	C	A	10:58	21:03	CRH380BL	1090
G5	A	B	06:15	09:05	CRH380BL	505
G6	B	A	10:55	13:05	CRH380BL	505
G7	A	B	07:45	10:11	CRH380BL	505
G8	B	A	11:26	14:02	CRH380BL	505
G9	A	B	13:55	17:00	CRH380BL	505
G10	B	A	18:32	21:25	CRH380BL	505
G11	A	B	14:50	18:00	CRH380BL	505
G12	B	A	19:16	22:00	CRH380BL	505
G13	A	B	19:28	22:58	CRH380BL	505
G14	B	A	06:50	09:10	CRH380BL	505
G15	B	C	06:10	09:30	CRH380BL	585
G16	C	B	19:26	22:45	CRH380BL	585
G17	B	C	07:54	10:25	CRH380BL	585
G18	C	B	06:55	09:58	CRH380BL	585
G19	B	C	10:30	13:55	CRH380BL	585
G20	C	B	08:10	11:20	CRH380BL	585
G21	B	C	11:40	14:50	CRH380BL	585
G22	C	B	15:10	18:17	CRH380BL	585
G23	B	C	18:57	22:10	CRH380BL	585
G24	C	B	16:28	19:38	CRH380BL	585
G25	B	C	20:27	23:28	CRH380BL	585
G26	C	B	17:57	21:02	CRH380BL	585

**Fig 4:**
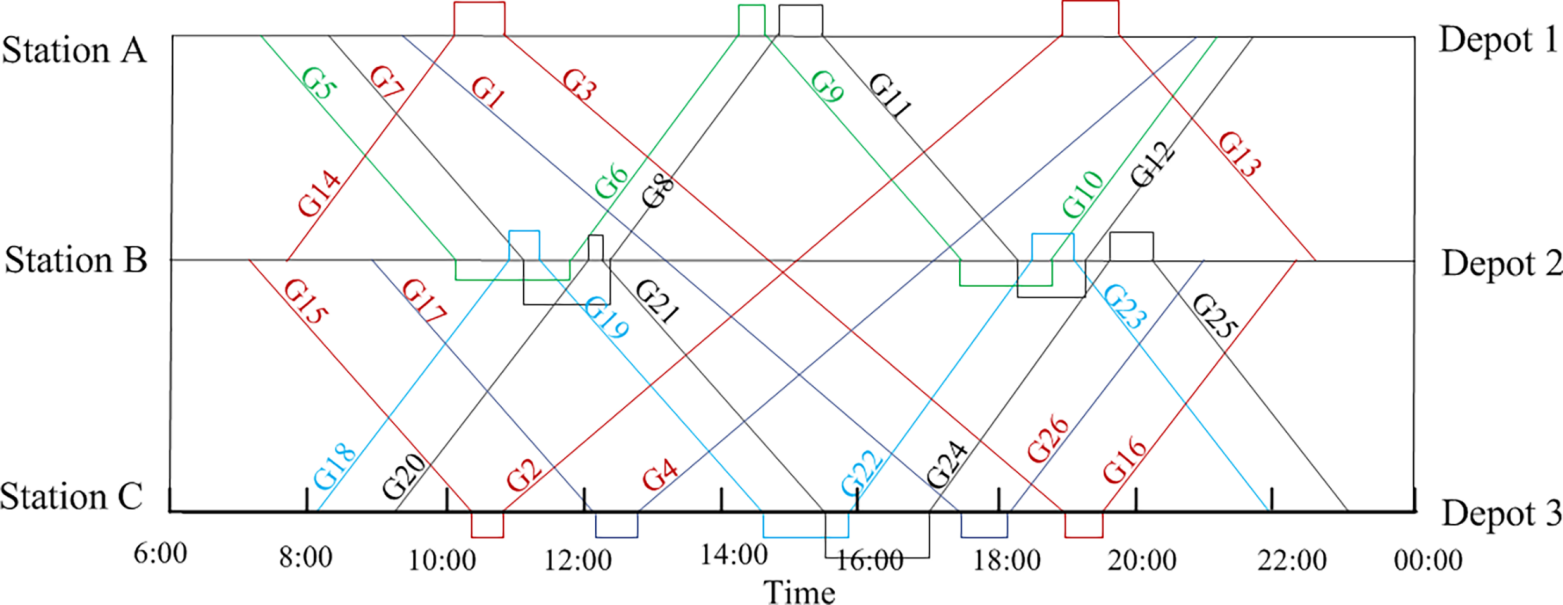
EMU circulation plan in the conventional mode based on the time-space diagram.

Fig 4 shows the optimization results calculated in the conventional mode. To facilitate observation, the connections between the trains are shown in Fig 4. An EMU circulation plan also can be expressed in the form of a train number chain, for example G5-G6-G9-G10. The specific routes in the EMU circulation plans shown in Fig 4 have been converted to Table 7, which shows the relevant information for each plan.

The EMUs return to their home depots for maintenance through the optimal train connection by forming a loop.

**Table 7:** EMU circulation plan.

EMU circulation plan number	Train connections	Maintenance depot	Total accumulated mileage (km)
1	G1-G26-G17-G4	Depot 1	3350
2	G18-G19-G22-G23	Depot 3	2340
3	G5-G6-G9-G10	Depot 1	2020
4	G15-G2-G13	Depot 2	2180
5	G14-G3-G16	Depot 2	2180
6	G7-G8-G11-G12	Depot 3	2020
7	G20-G21-G24-G25	Depot 3	2340

**Fig 5:**
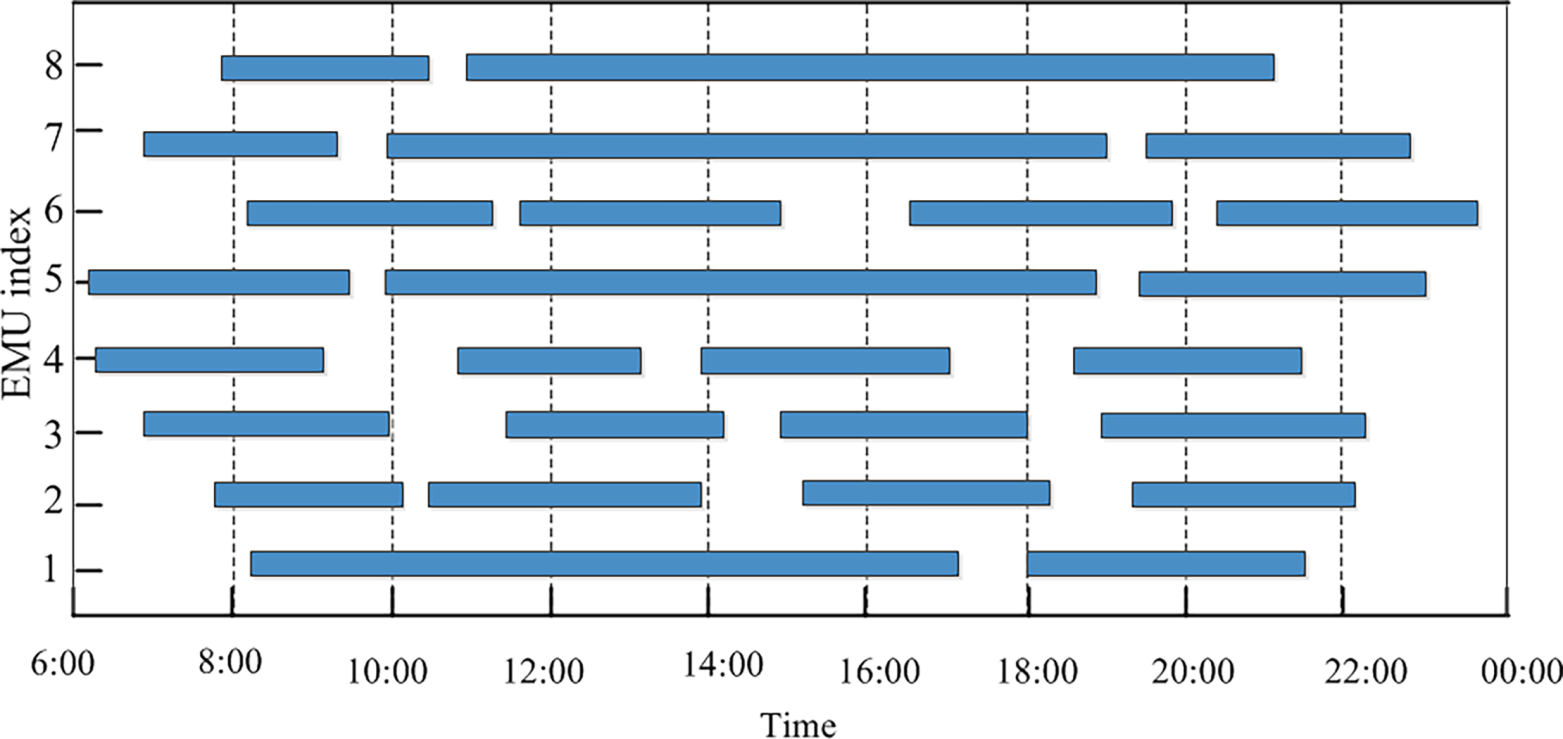
Gantt chart of the train tasks assigned to each EMU in the conventional mode.

We show the train tasks assigned to each EMU in Fig 4 as a Gantt chart in Fig 5; each row represents an EMU, the vertical axis is EMU index, and the horizontal axis is time. From the graph, we see that optimizing the connections among the trains has ensured optimal EMU utilization for each train. In this easy verified case, we see intuitively that from the perspective of any train, it is not possible to find other connections by decreasing the number of EMUs to improve the EMU utilization.

**Fig 6:**
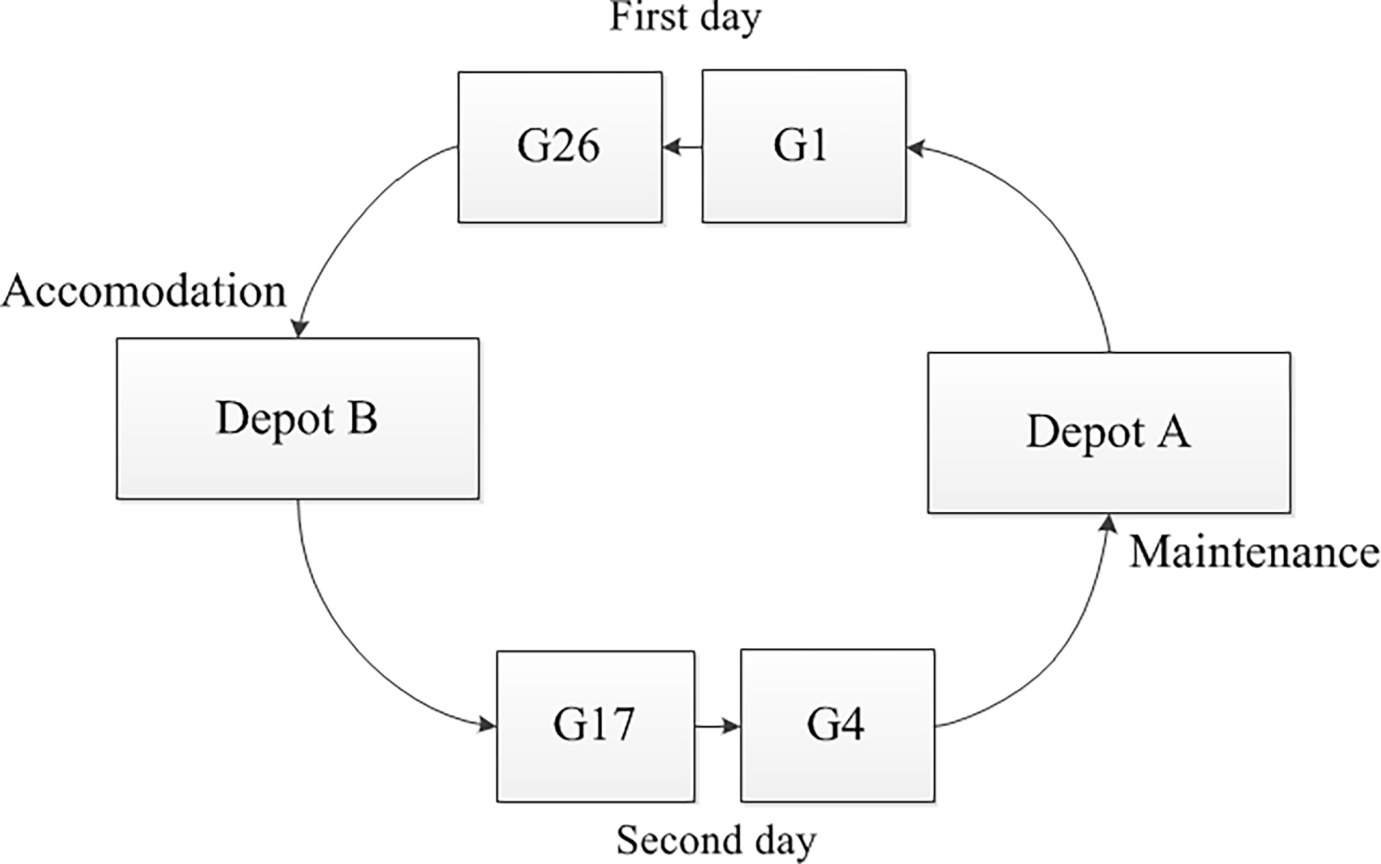
A two-day EMU circulation plan.

Fig 6 represents EMU circulation plan G1-G26-G17-G4, which lasts two days and in which two EMUs are required to cover the trains simultaneously. The two EMUs follow each other to form a circuit. During the cyclic process, each EMU is maintained when necessary rather than every day.

#### 6.1.2 EMU circulation planning in the collaborative mode

In the above-mentioned case, it is easy to satisfy the requirement to return for maintenance due to travel distance. Table 8 shows data obtained by modifying Table 6 slightly to make the train travel a longer distance. In this subsection, the new problem is to create an EMU circulation plan in accordance with the new train timetable subject to a limit of two EMUs being maintained at Depot 3.

**Table 8:** Train information for the case.

Train number	Origin station time	Destination station	Departure time	Arrival time	EMU type	Travel distance (km)
G1	A	C	08:08	17:02	CRH380BL	1120
G2	C	A	09:45	18:50	CRH380BL	1120
G3	A	C	09:55	19:02	CRH380BL	1120
G4	C	A	10:58	21:09	CRH380BL	1120
G5	A	B	06:15	09:12	CRH380BL	520
G6	B	A	10:55	13:12	CRH380BL	520
G7	A	B	07:37	10:11	CRH380BL	520
G8	B	A	11:26	14:09	CRH380BL	520
G9	A	B	13:55	17:07	CRH380BL	520
G10	B	A	18:32	21:32	CRH380BL	520
G11	A	B	14:43	18:00	CRH380BL	520
G12	B	A	19:16	22:07	CRH380BL	520
G13	A	B	19:20	22:58	CRH380BL	520
G14	B	A	06:50	09:17	CRH380BL	520
G15	B	C	06:10	09:37	CRH380BL	600
G16	C	B	19:19	22:45	CRH380BL	600
G17	B	C	07:54	10:33	CRH380BL	600
G18	C	B	06:48	09:58	CRH380BL	600
G19	B	C	10:22	13:55	CRH380BL	600
G20	C	B	08:10	11:27	CRH380BL	600
G21	B	C	11:32	14:50	CRH380BL	600
G22	C	B	15:10	18:24	CRH380BL	600
G23	B	C	18:57	22:17	CRH380BL	600
G24	C	B	16:20	19:38	CRH380BL	600
G25	B	C	20:20	23:28	CRH380BL	600
G26	C	B	17:57	21:09	CRH380BL	600

**Fig 7:**
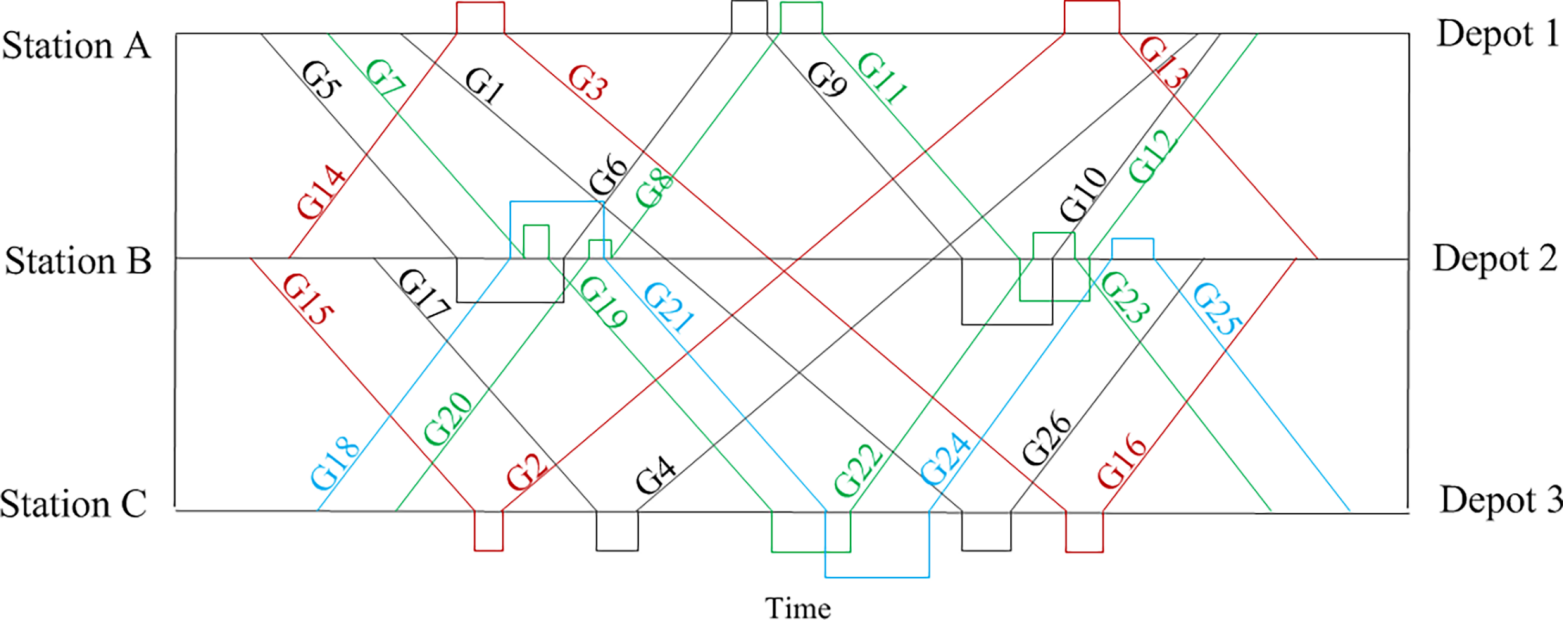
EMU circulation plans in the collaborative mode subject to the maintenance capacity limitation based on the time-space diagram.

**Fig 8:**
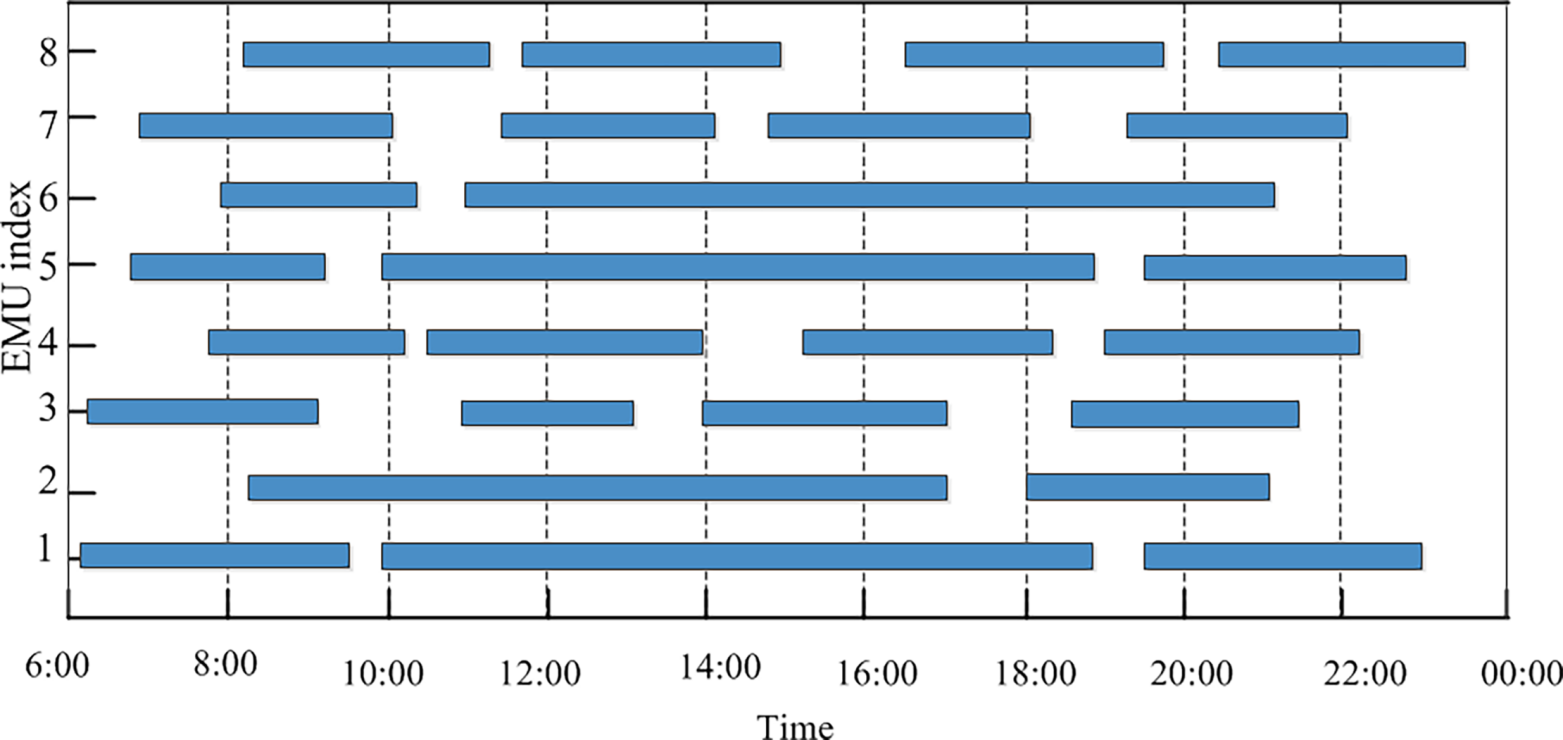
Gantt chart of the train tasks assigned to each EMU in the collaborative mode.

Fig 7 shows the EMU circulation plans in the collaborative mode subject to the maintenance capacity limitation. The situation in Fig 8 is similar to that of Fig 5. Further adjustments to the train connections cannot decrease the number of EMUs in order to improve EMU utilization.

**Fig 9:**
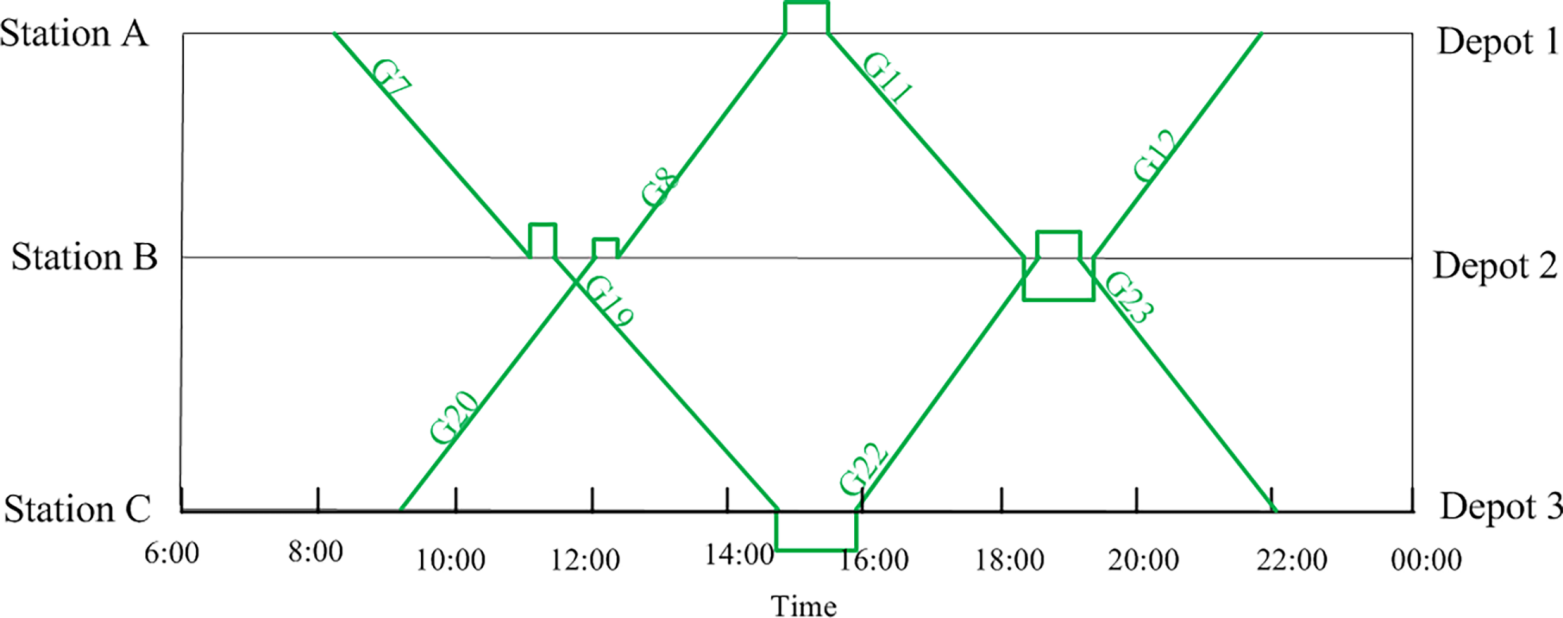
Specific EMU circulation plan based on the time-space diagram.

Table 9 shows the data from Fig 7 in the form of a table, and the results are reconstructed. For train G7-G22-G22-G20-G8-G11-G12, this can be used for off-site maintenance, and the specific process is shown in Fig 9. An EMU departs Depot 3 and is maintained at Depot 1, which is on its way, when it needs maintenance. The next day, the EMU operates along the route given in the EMU circulation plan and returns to its home depot, Depot 1, for maintenance. Releasing the maintenance location restrictions can produce more path combinations, which provides more optimization choices for actual problems. In the study below, the actual impact of the EMU maintenance management mode for a real case study is analyzed.

**Table 9:** EMU circulation plans in the collaborative mode.

EMU circulation plan number	Train connection	Maintenance depot	Total accumulated mileage (km)
1	G14-G3-G16	Depot 2	2240
2	G15-G2-G13	Depot 2	2240
3	G7-G19-G22-G23-G20-G8-G11-G12	Depot 1, Depot 3	4480
4	G1-G26-G17-G4	Depot 1	3470
5	G18-G21-G24-G25	Depot 3	2400
6	G5-G6-G9-G10	Depot 1	2080

### 6.2 Real case study

Due to the difficulty of solving the real problem when EMU maintenance restrictions are considered, this case study mainly analyzes and discusses the effects of different maintenance management modes.

#### 6.2.1 Line situation

The case of a real-world high-speed railway is used to study the effects of the maintenance mode in which the EMUs return home and the cooperative EMU maintenance mode on the EMU utilization efficiency. In addition, how the higher maintenance mileage limit, 5000 km, affects the EMU utilization efficiency is studied. Because there is a very high proportion of long-range high-speed trains traveling 1000–2000 km distances on the real-world high-speed railway, which is 2298 km long, the primary maintenance regulation is basically defined as 4000 km/48 h. This limits the EMU circulation plan because, according to statistics, most EMUs travel an average of 2500–3500 km or 3000–3500 km between maintenance stops on the real-world line. An optimization experiment is conducted that simultaneously considers the collaborative mode and increasing the maintenance mileage limit to 5000 km/48 h to improve the EMU utilization efficiency, which needs to be analyzed. For brevity, we refer to this as *collaborative mode with 5000 km*.

The train timetable under consideration contains 348 trains. The relevant depots, their EMU type restrictions, and the correspondences between depots and stations are given in Table 10.

**Table 10:** Real-world high-speed rail depots and services at high-speed rail stations.

Depot index	Depot	Station	Available EMU types
1	Beijing West Depot	Beijing West Station	CRH380A, CRH380AL
2	Shijiazhuang Depot	Shijiazhuang Station	CRH380A, CRH380AL, CRH5A
3	Guangzhou South Depot	Guangzhou South Station	CRH3C, CRH380BL
4	Shenzhen North Depot	Shenzhen North Station	CRH3C, CRH380BL
5	Changsha South Depot	Changsha South Station	CRH3C
6	Hongqiao Depot	Hongqiao Station	CRH380BL
7	Wuhan Depot	Wuhan Station	CRH2C, CRH380AL
8	Hankou Depot	Hankou Station	CRH2A, CRH5A
9	Zhengzhou East Depot	Zhengzhou Station	CRH380AL
10	Xi'an North Depot	Xi'an North Station	CRH2A, CRH2C, CRH380AL
11	Jinan Depot	Jinan Station	CRH380BL

#### 6.2.2 Optimization results

The real case represents the actual manual planning results. "Conventional mode," "collaborative mode," and "collaborative mode with 5000 km" refer to the optimization results obtained by the model and algorithm under different conditions.

(1) Analysis of the number of EMUs

The number of EMUs required in each maintenance mode is shown in Table 11.

**Table 11:** Number of EMUs used by each depot in each EMU maintenance management mode.

Depot	EMU type	Real case	Conventional mode	Collaborative mode	Collaborative mode with 5000 km
Beijing West Depot	CRH380AL	9	7	8	10
	CRH380A	10	10	9	9
Shijiazhuang Depot	CRH380AL	9	8	8	8
	CRH5A	2	2	2	2
Guangzhou South Depot	CRH380BL	9	9	9	8
	CRH3C	23	23	29	30
Shenzhen North Depot	CRH3C	6	6	2	4
Changsha South Depot	CRH3C	10	10	7	2
Hongqiao Depot	CRH380AL	15	16	15	15
	CRH380BL	4	4	4	4
Wuhan Depot	CRH380A	1	1	1	1
	CRH2C	7	7	7	7
Hankou Depot	CRH380AL	2	2	2	2
Zhengzhou East Depot	CRH380AL	15	15	15	15
	CRH5A	2	2	2	2
Xi'an North Depot	CRH380AL	5	5	5	5
	CRH2C	8	8	8	8
Jinan Depot	CRH380BL	1	1	1	1
Total EMUs	-	138	136	134	133

In the different EMU maintenance modes, the optimization process decreased the number of EMUs required. The number of EMUs required to cover the high-speed railway timetable gradually decreased, and the number of EMUs assigned to each depot changed because the connections between trains in the EMU circulation plan were optimized.

(2) Analysis of the EMU maintenance task results

1) Analysis of the number of EMU maintenance tasks and the average maintenance mileage

As shown in Table 12, the number of EMU maintenance tasks for each maintenance mode shows that the EMU maintenance was optimized to different degrees. In the cooperative maintenance mode, the number of maintenance tasks was decreased by 10.4%. In this mode, collaboration increased the number of possible combinations, which would allow more EMU circulation plans to be generated for further optimization. In the 5000 km mode, the number of EMU maintenance tasks decreased by 33.7%. In this mode, due to the mileage limit increase, EMUs can travel longer before requiring maintenance, which decreases the number of EMU maintenance tasks required. The above data show that using the collaborative EMU maintenance mode and raising the maintenance-mileage limitation can decrease the number of EMU maintenance tasks significantly.

**Table 12:** Number of EMU maintenance tasks in each EMU maintenance mode.

EMU maintenance mode	Number of EMU maintenance tasks	Average mileage before maintenance (km)	Percent decrease in the number of EMU maintenance tasks (%)
Real case	116	2842	**—**
Conventional mode	111	2970	-4.4
Collaborative mode	104	3169.9	-10.4
Collaborative mode with 5000 km	77	4281	-33.7

2) Analysis of the EMU maintenance tasks by depot

**Fig 10:**
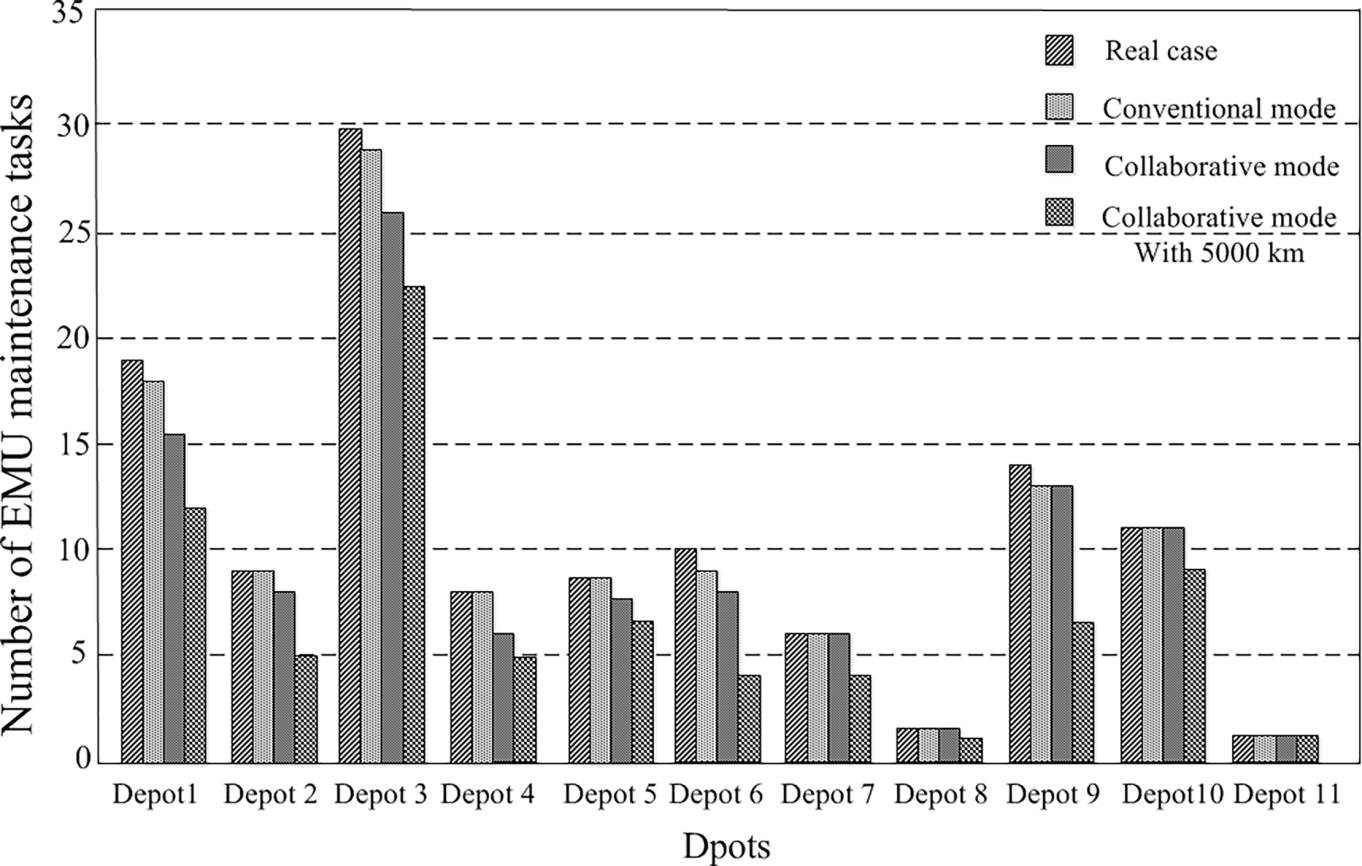
Number of EMU maintenance tasks at each depot.

Fig 10 shows the EMU maintenance tasks assigned to each depot for several EMU circulation plans. When the maintenance location restriction is relaxed, and the maintenance mileage limit is increased, most of the depots were found to have fewer EMU maintenance tasks to perform, but the Hefei South depot had more. There was an overall decreasing trend across depots, but individual depots showed anomalies. Because of the optimization requirements, part of the maintenance required at one location can be transferred to another to optimize the objectives.

3) Analysis of the changes in the number of EMU maintenance tasks within different EMU mileage ranges

Based on a statistical analysis, Table 13 shows the percentages of EMU maintenance tasks performed within certain mileage ranges divided by the number of EMU maintenance tasks. Under the different maintenance management modes, most of the EMU maintenance tasks were performed between 2000 and 3000 km; these accounted for 50% of the EMU maintenance tasks. Most of the EMUs were maintained every day. When the EMU maintenance mileage was set to 5000 km, the number decreased significantly; approximately 76% of the maintenance tasks were performed after the EMU had traveled 4000 km, and most of the EMUs were maintained every two days, which shows that increasing EMU maintenance mileage is an effective cost-saving measure.

**Table 13:** Distribution of maintenance tasks performed in different mileage ranges (%).

EMU maintenance mode	Maintenance mileage ranges (km)				
	1000–2000	2000–3000	3000–4000	4000–5000	>5000
Real case	3.8	53.8	32.1	10.3	0
Conventional mode	2.1	42.6	35.8	19.5	0
Collaborative mode	1.7	37.1	40.3	20.9	0
Collaborative mode with 5000 km	1.5	10.5	11.9	43.3	32.8

## 7 Conclusions

In this study, we have considered different maintenance modes and established an optimization model that simultaneously incorporates important constraints, including restrictions on EMU maintenance locations, limits on maintenance capacity and night accommodation capacity, and EMU type restrictions at depots. A branch-and-price algorithm is then used to solve the model. Taking a real-world high-speed rail as a case study, we optimized the circulation plans for different EMU maintenance management modes. The following conclusions can be drawn: through optimization, the number of EMUs can be decreased by optimizing the connections between trains in the EMU circulation plan; the maintenance workload can be decreased by optimizing the maintenance mode—the collaborative mode, in which maintenance resources are shared, decreased the number of EMU maintenance tasks required by the real-world high-speed railway by 12.3%; sharing maintenance resources and increasing the maintenance mileage limit to 5000 km are significant because the results showed that the number of EMU maintenance tasks was decreased by 36.8%, which released a significant amount of time previously allocated to EMU maintenance tasks. This study provides high-speed railway operators with a helpful decision-making tool for improving EMU utilization efficiency.
